# Effects of the lifestyle habits in breast cancer transcriptional regulation

**DOI:** 10.1186/s12935-016-0284-7

**Published:** 2016-02-13

**Authors:** Marco Allán Pérez-Solis, Guadalupe Maya-Nuñez, Patricia Casas-González, Aleida Olivares, Arturo Aguilar-Rojas

**Affiliations:** Research Unit in Reproductive Medicine, Hospital de Ginecobstetricia “Luis Castelazo Ayala”, Instituto Mexicano del Seguro Social, No. 289 Río Magdalena, Tizapan San Angel, 01090 Mexico, DF Mexico

**Keywords:** Breast cancer, Transcription, Obesity, Ethanol, Tobacco

## Abstract

Through research carried out in the last 25 years about the breast cancer etiology, it has been possible to estimate that less than 10 % of patients who are diagnosed with the condition are carriers of some germline or somatic mutation. The clinical reports of breast cancer patients with healthy twins and the development of disease in women without high penetrance mutations detected, warn the participation more factors in the transformation process. The high incidence of mammary adenocarcinoma in the modern woman and the urgent need for new methods of prevention and early detection have demanded more information about the role that environment and lifestyle have on the transformation of mammary gland epithelial cells. Obesity, alcoholism and smoking are factors that have shown a close correlation with the risk of developing breast cancer. And although these conditions affect different cell regulation levels, the study of its effects in the mechanisms of transcriptional and epigenetic regulation is considered critical for a better understanding of the loss of identity of epithelial cells during carcinogenesis of this tissue. The main objective of this review was to establish the importance of changes occurring to transcriptional level in the mammary gland as a consequence of acute or chronic exposure to harmful products such as obesity-causing foods, ethanol and cigarette smoke components. At analyze the main studies related to topic, it has concluded that the understanding of effects caused by the lifestyle factors in performance of the transcriptional mechanisms that determine gene expression of the mammary gland epithelial cells, may help explain the development of this disease in women without genetic propensity and different phenotypic manifestations of this cancer type.

## Resumen

A través de la investigación realizada en los últimos 25 años en torno a la etiología del cáncer de mama, ha sido posible estimar que menos del 10 % de las pacientes que son diagnosticadas con la enfermedad son portadoras de alguna mutación de línea germinal o somática. Los informes clínicos de pacientes de cáncer de mama con gemelas saludables y el desarrollo de la enfermedad en las mujeres sin mutaciones de alta penetrancia detectadas, advierten la participación de otros factores en el proceso de transformación. La alta incidencia de adenocarcinoma de mama en la mujer moderna y la necesidad urgente de nuevos métodos para la prevención y detección temprana han exigido una mayor información en relación al papel que el medio ambiente y el estilo de vida tienen en la transformación de las células epiteliales de la glándula mamaria. La obesidad, el alcoholismo y el tabaquismo son factores que han demostrado una estrecha correlación con el riesgo de desarrollar cáncer de mama. Y aunque estas condiciones pueden afectar distintos niveles de regulación celular, el estudio de sus efectos en los mecanismos de regulación transcripcional y epigenética, es considerado fundamental para un mayor entendimiento de la pérdida de identidad de las células epiteliales durante la carcinogénesis de este tejido. El objetivo principal de esta revisión fue establecer la importancia de los cambios que ocurren a nivel transcripcional en la glándula mamaria, como consecuencia de una exposición aguda o crónica a productos nocivos, tales como los alimentos que favorecen la obesidad, el etanol y los componentes del humo del cigarro. Al analizar los principales estudios relacionados con el tema, se ha llegado a la conclusión de que la comprensión de los efectos causados por los factores de estilo de vida sobre el desempeño de los mecanismos de regulación transcripcional responsables de la expresión génica de las células epiteliales de la glándula mamaria, pueden ayudar a explicar el desarrollo de esta enfermedad en las mujeres que no son genéticamente propensas así como las diferentes manifestaciones fenotípicas de este tipo de cáncer.

## Background

The ethnic and regional variations reported in the incidence of breast cancer, as well as the sustained increase in the number of cases that this disease has since the 80s to date, are some topics that can not be explained solely in function of inherited or acquired mutations in high penetrance genes [1]. Epidemiological studies have reported a positive correlation between increased risk of breast cancer and the growing consumption of high-fat foods, ethanol and tobacco by current women [2]. This has prompted a further research of the molecular mechanisms underlying the appearance of breast cancer by some habits that women have been adopting their lifestyle.

The oncogenic pathologies are currently considered the result of the loss of cell identity caused by the disturbance in expression of proteins associated with the cell differentiation. Among the multiple factors that can alter gene expression programs, the high penetrance mutations, inherited or acquired, are the most easily detectable causes. However, the percentage of breast cancer cases etiologically associated with this type of mutations does not exceed 10 % [3, 4]. The high probability of developing cancer from these mutations is explained by the fact that the affected genes, encode proteins involved in cellular processes such as: DNA repair, cell cycle regulation and protection against xenobiotics and free radicals, which are essential functions for maintaining the fidelity and integrity of the genome [4]. In the case of breast cancer, genetic linkage analysis and next generation sequencing studies have found that higher penetrance mutations occur mainly in the genes encoding: breast cancer 1 (BRCA1), tumor protein p53 (TP53), phosphatidylinositol-4,5-bisphosphate 3-kinase catalytic subunit alpha (PIK3CA), retinoblastoma 1 (RB1), phosphatase and tensin homolog (PTEN), GATA binding protein 3 (GATA-3) and mitogen-activated protein kinase kinase kinase 1 (MAP3K1) [5–7]. Such modifications commonly alter the open reading frame of the gene, resulting in the loss or gain of function in the encoded proteins [4]. However, the involvement of mutations in regions of gene transcriptional regulation, and its association in the development of breast cancer needs further investigation.

On the other hand, the association that the epidemiology studies have found between the process of cell malignancy and some lifestyle factors such as smoking, diet, alcoholism and obesity; has prompted a further research on the role of alterations produced by these factors in the mechanisms of gene expression regulation [2]. Although there are several regulation levels for the gene expression, the transcriptional regulation mechanisms are considered the fundamental system whereby the cells can modulate, halt or activate the expression of a given gene [8]. So that any malfunction or modification in the expression of the proteins involved in these mechanisms could have important consequences in the cell protein expression.

### Relationship between lifestyle risk factors and breast cancer

The molecular biology studies have provided evidences for cancer etiology and now it is known that genetic mutations occur constantly and naturally in the cells, as a collateral consequence of the processes of recombination, replication and cell metabolism [9, 10]. There are enzymatic systems responsible for the prevention, repair and correction of damage or errors caused for the normal cell operation. However, in cancer cells the stress induced by disease itself or by external factors can increase the mutation frequency, since protection and proofreading systems are overcome by high free radical levels characteristic of these conditions [3, 11]. Certain chemical agents, ionizing radiations and even poorly balanced diets are currently considered risk factors that may contribute to cancer development [11, 12]. These factors can induce cell proliferation, survival and metastasis through either gene mutations on the proteins responsible for cell protection and maintenance, or by its molecular interaction with proteins involved in transcriptional regulation mechanisms [13].

One explanation to the increase in the incidence of breast cancer it has been based precisely on the growing trend of women to adopt certain consumption habits, such as the high-fat diets, uptake of alcoholic beverages and tobacco, which are risk factors for this illness [13]. The overconsumption of fat foods, for example, increases both the hyperplasia and hypertrophy of adipose tissue. This tissue is the main producer of estrogens in post-menopausal women, so that their uncontrolled growth leads to greater exposure than normal to estrogens during the life of the woman. The contribution of these hormones on cell proliferation increases the probability that those cells carrying high penetrance mutations thrive within the mammary gland [14–16].

On the other hand, the acetaldehyde is the main catabolite of the ethanol metabolism in humans and has been classified as a carcinogen by the International Agency for Research on Cancer [17]. This compound is capable of forming adducts with DNA, promoting the process of mutagenesis and cell malignancy [18]. In addition, clinical studies have reported a correlation between moderate ethanol intake (15–30 g/day) and increased risk of breast cancer in the premenopausal women group homozygous for the alcohol dehydrogenase 3^1^ alelle (ADH_3_^1−1^); phenotype that be characterize by an activity increased of alcohol dehydrogenase and thus a major production of acetaldehyde from ethanol [19].

Regarding the role of smoking on carcinogenesis in the breast tissue; some clinical and epidemiological studies have found a strong correlation between women with active exposure to tobacco smoke and breast cancer incidence [20, 21]. Furthermore, the presence of various chemicals generated by the cigarette combustion, have been detected in mammary gland tissue of healthy women and breast cancer patients [22–24]. On the other hand, evidence from in vitro studies suggest that various substances from the cigarette smoke such as nicotine [25], benzo(a)pyrene [26], 1-methylanthracene [27] and phenanthrene [28] may to provoke alterations in epigenetic and transcriptional mechanisms which regulates the expression of genes involved in transformation of healthy epithelial cells as well as proliferation and metastasis of tumor epithelial cells into the mammary gland.

### Transcriptional regulation in breast cancer

The progressive loss of cell morphology and function are events that commonly occurs among the different types of cancer, as a consequence of the drastic changes in gene expression of cells affected during the disease development. However, studies performed in reference to the etiology underlying to cancer, indicate that triggering causes of this transformation are diverse and may have an epigenetic, genetic or post-translational origin [29]. These alterations can affect the expression or function of certain proteins, such as transcription factors, co-regulators, histones, as well as enzymes that modify histones and DNA; each of which play a specific role in the mechanisms of transcriptional regulation [3, 30, 31].

The overexpression and gene amplification of transcription factors, such as estrogen receptor alpha (ERα) [31], avian myelocytomatosis viral oncogene homologue factor (c-myc) [32] are among first alterations of the transcriptional regulation mechanisms that were found associated to development of breast cancer. At present, due to its role in proliferation of the mammary adenocarcinoma cells, are considered key oncoproteins for diagnosis for this pathology. Conversely, tumor suppressor proteins, such as retinoblastoma protein (Rb) [33] and p53 [32], are common targets of different mutation events that eliminate the repressive function of these transcription factors over genes associated with the replication and cell division in malignant breast tumors.

Several research lines have provided evidence of that a select group of transcriptional regulators, termed as “Master”, work together in the trans-activation of critical genes for maintaining of plasticity and unlimited propagation that characterize to the embryonic stem cells [29, 30, 34]. However, it has also been reported that others cell types differenced or malignant, also have a cell-specific repertory of Master transcription factors that defines its gene expression scheme. Even recently been shown that Master factors can exercise its transcriptional activity on cell-identity genes or oncogenes, through its interaction with the Mediator multiprotein coactivator and regulation regions sets, known as Super-enhancers [29, 35]. This new mechanism increases stability and processivity of the general transcription machinery, thereby increasing the transcription of target genes. These findings confirm the crucial role that transcriptional control mechanisms have in the cellular changes during development, as well as the vulnerability of these same mechanisms to the alterations that trigger the transformation of healthy cells to malignant cells.

Recent analyzes of reporter activity arrays of transcription factors in different breast cancer-derived cell lines, have found more specifically that Master factors Twist, Snail, Slug and E47 are involved in the establishment of some phenotypic processes, such as epithelial-mesenchymal transition and metastasis [36, 37]. Furthermore, Martin and colleagues have found previously a correlation between increased expression of these Master factors and breast cancer tumors with metastasis or poor prognosis [38]. In addition it has also shown that these same factors may repress the transcription of gene CDH1 encoding cadherin-1 (Cadh-1), through the interaction of its C2H2 type zinc finger domain with E boxes located within promoters regulating the expression of this protein that is important for adhesion and polarity of mammary gland epithelial cells [39].

Regarding the role of Super-enhancers in development of breast cancer, it has been reported that the oncogenes: human epidermal growth receptor 2 (ERBB2), hepatocyte growth factor receptor (MET), MYC, nuclear receptor coactivator A3 (NCOA3), neurogenic locus notch homolog protein 2 (NOTCH2) and Runt-related transcription factor 1 (RUNX1) are part of specific expression scheme that is induced by this new regulatory regions for the acquisition of the malignant phenotype [30]. Importantly, the last four genes encode proteins with functions closely associated with the regulation of transcription. This suggests that the identity of the breast cancer cells could be controlled by an hierarchical regulation organization in where the top of the pyramid is occupied by Master transcription factors that trans-activate the expression of other factors of lower hierarchy but with a higher cellular specificity; that in turn regulate the transcription of genes involved in the expression of the final phenotypic characteristics.

### Effects of obesity in transcriptional regulation

**Fig. 1:**
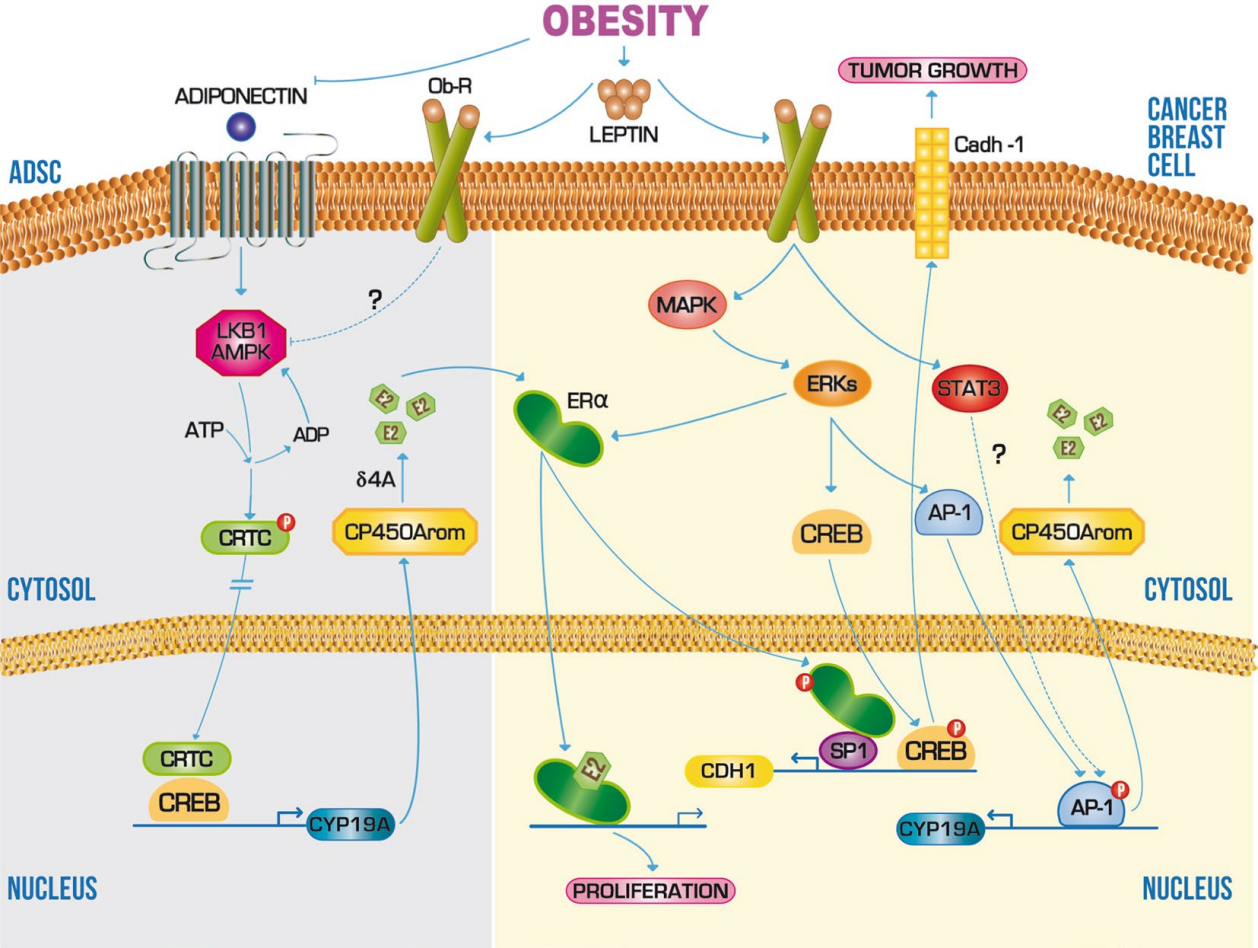
Scheme of molecular mechanisms that obesity can influence during carcinogenesis of the mammary gland. The abnormal increase of leptin levels in obesity is an event which can positively regulate transcription of genes associated with tumor growth and proliferation such as Cadh-1 and CP450Arom, respectively. *Solid lines* indicate a mechanism or pathway established experimentally. *Dashed lines* indicate a mechanism or pathway not determined yet. *Arrowheads* indicate upregulation and* flattened heads* indicate downregulation

Evidence of the proliferative effect that obesity has in health and malignant epithelial cells in the mammary gland is extensive. It is known that this effect is due to an imbalance in levels of adipose-derived hormones to regulate the energy metabolism [40–43]. During the post-menopause, women stop the production of ovarian estrogen, leaving to the adipose tissue as the main supplier of this hormone. When the energy reserves exceed the storage capacity of this tissue, the adipocytes undergo both hyperplasia and hypertrophy which result in increased levels of Leptin, one major hormones produced by adipocytes that travel through the bloodstream to the hypothalamus to induce the satiety signal. However, it is known that the leptin receptor (Ob-R) is expressed in other cell types, such as the adipose derived stromal cells (ADSCs) and epithelial cells of the mammary gland; in where the leptin acts in paracrine and autocrine way for the induction of several signaling pathways converging in the induction of cell proliferation [44]. For purposes of this review, will be addressed three of these pathways, which exemplify widely the relevance of the transcriptional regulation mechanisms in the development of malignant tumors in the mammary gland (Fig. 1).

The ADSCs are an adipose tissue cell type with the capacity to synthesize and secrete 17-β estradiol (E2) through enzymatic aromatization of δ-4-androstenedione (δ4A) by cytochrome P450 aromatase (CP450Arom). Because of the importance of the E2 in activation of several genes of cell proliferation, it is considered that the transactivation of cytochrome P450, family 19, subfamily A (CYP19A) gene, is a critical control point for the formation and survival of malignant tumors expressing estrogen receptor alpha (ERα) [45]. One mechanism considered as key to the aromatase mRNA expression is the recruitment of cAMP response element binding protein (CREB) to the proximal promoter of CYP19A (called PII). For CREB to carry out the CYP19A transactivation, this transcription factor must first bind one of its main coactivators, the CREB regulated transcription coactivator (CRTC) [40]. The nuclear translocation and activity of the CRTC is in turn modulated by phosphorylation of its Ser-171 residue via adiponectin receptor (AdipoR)/liver kinase B1 (LKB1)/AMP activated protein kinase (AMPK) signaling pathway. Adiponectin is other adipokine that under physiological conditions its serum concentration is higher than leptin, allowing modulate to the CP450Arom and E2 levels in ADSCs [46]. Conversely, the uncontrolled secretion of leptin caused by obesity, inhibits the phosphorylation of CRTC through Ob-R, increasing thus the CP450Arom expression and local production of E2 (Fig. 1). Consequently, the increase of E2 levels in the mammary gland, amplifies the expression of genes associated with cell proliferation in breast ductal epithelium [41].

On the other hand, in the mammary gland epithelial cells, leptin stimulate the production of Cadh-1, a protein that is used by the epithelial cells for the formation of adherent junctions (Fig. 1). The role of Cadh-1 has been experimentally correlated, with both growth of early primary breast carcinoma and metastasis suppression of most advanced tumors [47]. In addition, others in vitro studies have found that the interaction of leptin with Ob-R activates the extracellular signal-regulated protein kinases (ERKs) pathway, which in turn induces the nuclear translocation and binding of CREB and ERα to cAMP response element (CRE) and specific protein 1 (SP1), respectively, in the CDH1 promoter [48]. Leptin-induced interaction between ERα and SP1 is independent of E2, so that can be inferred that leptin enhances the non-classical genomic pathway of ER in the transcriptional activation mechanism of Cadh-1 [49].

Studies in cell lines derived from breast cancer have shown that malignant epithelial cells, also induces expression functional of CP450Arom through leptin and its receptor [41–43]. Although the mechanism of transcriptional activation of CYP19A in malignant cells is not completely understood, it is known that the CYP19A promoter is transactivated through its *cis*-element for activating protein 1 (AP1) in the cell line derived from breast cancer MCF 7 (Fig. 1). Electrophoretic mobility shift assays in the presence of leptin suggest that the transcription factor AP-1 could be activated through phosphorylation of mitogen activated protein kinase (MAPK) and/or the ERKs. Likewise, it was showed the involvement of signal transducer and activator of transcription 3 (STAT3) in transactivation of the AP-1 element, although it is still unknown at what level of mechanism this is involved [41]. Together these data indicate that leptin may induce directly E2-dependent cell proliferation of malignant epithelial cells in the mammary gland, through an alternative route that has not been fully investigated.

### Effects of alcoholism in transcriptional regulation

The positive correlation that the epidemiological studies have found between moderate or chronic ethanol consumption and the incidence of breast cancer in pre- and post-menopausal women indicates the importance that environmental factors have in the development of this pathology [50–52]. Alcoholism, like other environmental risk factors, may cause alterations in gene expression through different pathways. Ethanol concentrations as low as 0.06 % have effect in the transcriptional expression of genes related with malign proliferation of mammary gland epithelial cells [53, 54]. Due to this characteristic it was considered relevant to review the molecular mechanisms through which the ethanol exerts its activity and influences the transcriptional regulation.

Because about 75 % of breast cancer patients are ERα-positive and the contribution of ethanol in estrogen-dependent induction of cellular proliferation, survival and metastasis [54, 55] has been demonstrated, the mechanisms underlying the increase of ethanol-induced estrogen activity have gained interest in both clinical and basic research. As previously mentioned, the over-expression of CP450Arom and ERα is usually critical to early development of mammary adenocarcinoma [53, 56]. In vitro studies have shown that moderate doses of ethanol induce the synthesis of ERα mRNA and the proliferation of breast cancer cells; however, the complete mechanism of transcriptional regulation remains elusive.

**Fig. 2:**
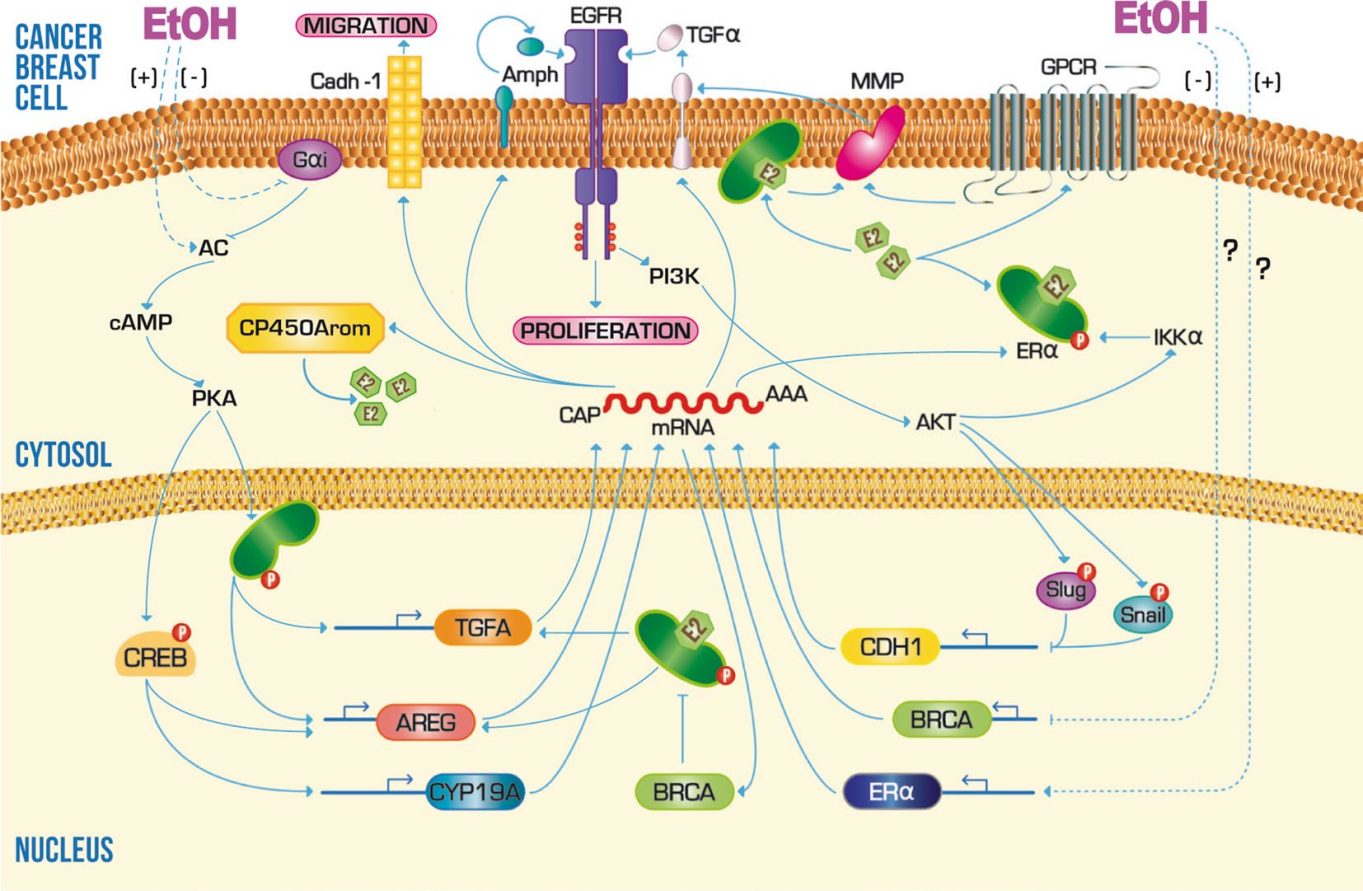
Scheme of the molecular mechanisms that ethanol consumption can influence during carcinogenesis of the mammary gland. Trace amounts of ethanol can promote transcription of CP450Arom, ERα and some EGFR agonists, which in turn favor the progress and survival of breast cancer cells. Acute exposure to ethanol can also negatively interfere the transcriptional regulation of genes that prevent the proliferation and spread of breast cancer, such as BRCA1 and Cadh-1. *Solid lines* indicate a mechanism or pathway established experimentally. *Dashed lines* indicate a mechanism or pathway not determined yet. *Arrowheads* indicate upregulation and* flattened heads* indicate downregulation

On the other hand, the investigation of the effects of ethanol upstream of the CP450Arom transcription found that acute and chronic exposure to ethanol concentrations above 0.5 % provides a greater stability to adenylyl cyclase (AC), blocks the Gαi function and promotes an irregular increase in cAMP production [54, 57–59]. This increase in cAMP levels in turn promotes the transactivation of target genes for CREB, such as CP450Arom [53] and the mitogen agonist belonging to the endothelial growth factor family, Amphiregulin (Amph) [48, 60, 61]. In breast cancer, it has been reported two coupling alternate routes of the E2 synthesis with EGFR signaling (Fig. 2). The first and most widely described is the ERα-dependent transcriptional activation pathway for the Amph and TGFα expression [60, 62–64]. Secondly, is the overstimulation of EGFR signaling pathways through membrane-bound classic estrogen receptors or G protein coupled estrogen receptor, which activate to the metalloproteinases responsible for release of EGFR agonists anchored to the cell membrane [65, 66]. There is also evidence obtained from a study of the breast cancer signaling pathways, which indicate that EGFR can induce to the ligand-independent and dependent activity of ERα by means of the protein kinase A (PKA) and inhibitor of kappaB kinase α (IKKα) phosphorylation, respectively, thereby closing the positive feedback loop [67, 68].

The existing bidirectional feedback between EGFR signaling and estrogenic activity is essential for the preservation of malignant phenotype in the breast cancer cells; since both pathways converge in the transactivation of genes correlated with the tumor proliferation, survival, aggressiveness; such as MYC [69, 70], B cell lymphoma 2-like protein (Bcl-XL) [71, 72], cyclin D1 (CCND1) [73, 74], cyclin-dependent kinase inhibitor A1 (CDKN1A) [75, 76] and the subunits that integrate the activator protein 1 (AP1) [77, 78]. However, recent studies of cell migration performed in the cell line of breast cancer MCF-7 have reported that ethanol, unlike to mechanism induced by leptin in obesity, can cause increased cell migration at a concentration of 12 %, an effect that was correlated mainly with a decrease in the expression of the Cadh-1 [48, 61]. Although not known for sure the mechanism by which ethanol inhibits expression of Cadh-1, there is evidence of recruitment of Master repressor factors, Snail and Slug, to the CDH1 promoter in breast cancer cells (Fig. 2). These two factors are activated directly or indirectly by ERK, AKT and p38; which are targets of EGFR activity [79]. So it would be very important to establish experimentally whether ethanol is involved in this mechanism to promote cell migration.

Furthermore, the repression of ER transcriptional activity by the interaction with BRCA1 is another event considered critical in the control of the mammary adenocarcinoma cells proliferation [80–82]. With the aim of determine if ethanol has any effect in this mechanism, it has recently been analyzed the mRNA and protein expression of both factors in ERα-negative cell lines and human epidermal growth factor receptor 2 (HER2)-positive mice tumors under treatment of increasing concentrations of ethanol. The results showed that under these conditions (Fig. 2), ERα levels increase in a dose–response way with respect to controls without treatment, while the BRCA1 levels decrease inversely proportional to the ethanol dose [80]. Furthermore, it was found that these alterations in the expression of both proteins are closely correlated with cell proliferation promoted by ethanol [55]. Although these results suggest the direct participation of ethanol on the transcriptional regulation mechanisms of genes encoding to ERα and BRCA1, further research is need.

Several clinic and epidemiological studies performed in humans and other mammalians suggest that some chronic disorders elicited by ethanol abuse, such as hepatosteatosis [83], megaloblastic anemia [84], pancreatic disorders [85] and certain cancers [66], are clinically associated with a systemic deficiency of folate. It has been shown that ethanol can interfere both the absorption and assimilation of this vitamin, thus affecting the normal synthesis of methionine by organism cells [86, 87]. This insufficiency in the methionine requirements induces a reprogramming on the genome activity, causing the expression of oncogenes in the cells [83, 88, 89]. Although this same mechanism has been implicated by some authors in the ethanol-induced rehabilitation of ERα expression in breast cancer [90], more molecular studies to support this idea are required. On the other hand, it has been shown that ethanol-induced global hypomethylation can alter both the expression and activity of DNA methyltransferases during carcinogenesis. This mechanism has been involved in the ethanol-induced specific hypermethylation of tumor suppressor genes [91]. However, the possibility that this mechanism explain the BRCA1 silencing observed in breast cancer cells treated with ethanol, not yet been explored.

### Effects of smoking in transcription regulation

Due to the large amount of compounds generated from the cigarette combustion, the search concerning the etiology and causal agents of breast cancer associated with smoking has been difficult. As mentioned previously, many of the effects caused by the tobacco are attributed to somatic mutations produced by adducts formed in genes of high penetrance that lead to cell malignancy. However, in vitro studies focused on gene expression changes between tumor and healthy cells from the mammary gland epithelium exposed to cigarette smoke extract (CSE); have shown that a large number of genes are regulated either positively or negatively. Nonetheless, the reproducibility of each phenotype obtained, even those with significant changes, was low [92]. Among the genes that were found with significant changes in expression, only ocludin and claudin-1 had an increase in its methylation; which is consistent with the previously observed significant decrease in their levels of mRNA and protein after CSE treatment. Additionally, it was found that the gene encoding estrogen receptor beta (ERβ) also had a higher methylation after CSE treatment [92]; which agrees with others reports of tumor suppression by this receptor [93, 94]. However, the specific compound in cigarette smoke and the mechanism through which triggers methylation and therefore epigenetic silencing of these genes, is still unknown.

**Fig. 3:**
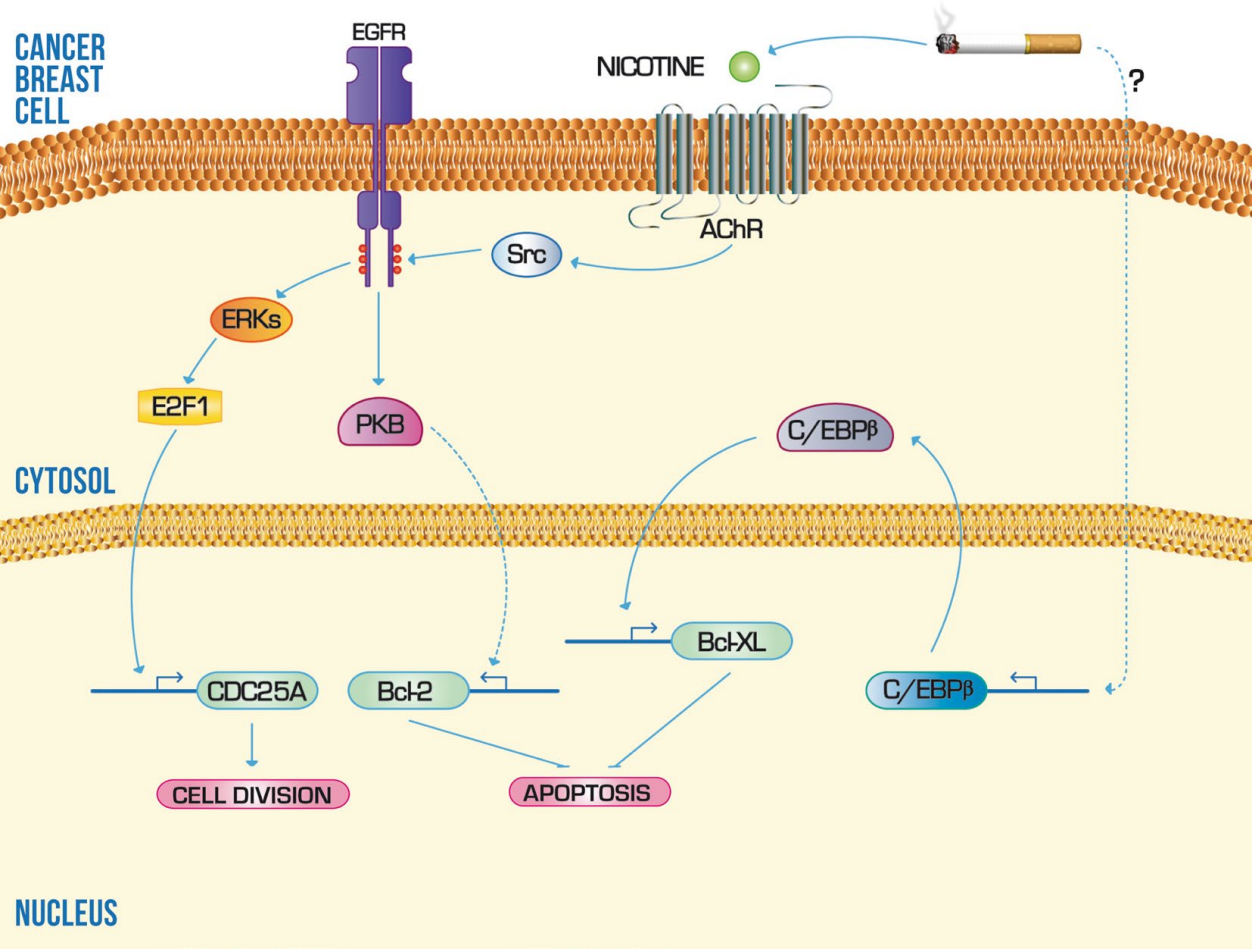
Scheme of the molecular mechanisms that systematic exposure to cigarette smoke can influence during carcinogenesis of the mammary gland. Different components of cigarette smoke can induce mechanisms, at transcriptional level, that promote the expression of anti-apoptotic and mitotic checkpoints genes. *Solid lines* indicate a mechanism or pathway established experimentally. *Dashed lines* indicate a mechanism or pathway not determined yet. *Arrowheads* indicate upregulation and* flattened heads* indicate downregulation

The transcriptional mechanisms that mediate the transformation and survival of malignant breast cells as a result of smoking have been studied. Connors et al., provided evidence supporting the participation of transcription factor CCAAT element binding protein beta (C/EBPβ), in the transactivation of anti-apoptotic gene Bcl-XL [95]. It is well established that the protein encoded by this gene is actively involved in the suppression of apoptotic caspase pathway (Fig. 3), and it is currently used as a marker of migration and tumor aggressiveness [96]. The exposure of non-malignant MCF-10A cell line to increasing concentrations of a cigarette smoke condensate (CSC) resulted in a rising in the expression of both mRNA and protein of Bcl-XL. It was further determined that the CSC simultaneously induces the C/EBPβ expression; which in turn interacts functionally with its *cis*-element in the Bcl-XL, and promotes thus the transcriptional activity in this gene [95]. Although the mechanism by which cigarette smoke increases C/EBPβ levels in these cells is not known yet, it is clear that smoking may induce the survival and aggressiveness of breast epithelial cells with high penetrance mutations.

Another molecular model that explain the relationship between the phenotypic features of breast tumor cells and smoking, was also partially solved from the nicotine effects in the transcriptional regulation Bcl-2 and the cell cycle-regulatory CDC25A phosphatase (Fig. 3). The nicotine is a compound of the cigarette smoke composition and was found to interact with the ionotrópic acetylcholine receptor (AChR) of nerve cells [97]. However, it has been reported that epithelial cells of others tissues also express the AChR for nicotine [98–100]. Assays performed in the cell lines MCF-10A and MDA-MB231 demonstrated that the nicotine may induce the simultaneous activity of ERKs and protein kinase B (PKB) by a crosstalk between the nicotinic AChR and EGFR, where the Scr kinase performs the connection. The ERKs pathway culminates in the phosphorylation of E2F1, which is a highly specific transcription factor of gene encoding CDC25A. On the other hand, the PKB phosphorylation pathway is responsible for inducing the transcriptional expression of Bcl-2, although it is unknown the *cis*-elements or factors involved [101].

## Conclusion

The research conducted around the identification of high penetrance mutations through the recent massive sequencing technologies, has facilitated the identification of target genes associated with each type of cancer. However, the etiologic contribution of genetic alterations to cancer development represents barely one-tenth of all possible causes [102]. In the case of breast cancer, several environmental and lifestyle factors have already been correlated with this disease through meta-analysis [1]; which has helped to narrow the search for the mechanisms involved at the molecular level in the transformation of the epithelial cells or tumor progression into mammary gland.

Much of the effects caused by main risk factors of lifestyle are associated in some way with estrogen-dependent molecular mechanisms that induce the proliferation and survival of cells in the breast cancer. However, in the particular case of tobacco, although the correlation between abusive consumption of this product and estrogen levels in post-menopausal women is well established [103], the mechanism by which this habit could be altering estrogen synthesis are still unknown. On the other hand, the investigation of estrogen-dependent molecular mechanisms that are altered by systematic intake of ethanol and fatty acids during malignant transformation in the mammary gland, has achieved an improvement in providing the fundaments that will allow a better understanding of the disease for further prevention and treatment.

The biological activity of acute or chronic consumption of fatty acids, ethanol and cigarette smoke, has been functionally linked to transcriptional processes that regulate the gene expression patterns of target cells in the mammary gland through leptin, EGFR family agonists and nicotine, respectively. However, it is important to note that some events such as leptin-dependent transactivation of CYP19A in breast cancer cells, changes induced by ethanol in transcriptional expression of ERα and BRCA1, and nicotine-dependent transcriptional induction of Bcl-2 are just some of the mechanisms that remain partially unclear and therefore require further investigation to help the integration of the molecular models that explain the intervention of high risk habits in transcriptional regulation during carcinogenesis of breast tissue.
